# Oocyte-specific knockout of eIF2 subunits causes apoptosis of mouse oocytes within the early growing follicles via mitochondrial dysfunctions and DNA damage

**DOI:** 10.1038/s41419-026-08449-y

**Published:** 2026-02-02

**Authors:** Huiyu Liu, Weiyong Wang, Biao Li, Shuang Liu, Hongwei Wei, Wenjun Zhou, Tiantian Hao, Ying Wei, Xiaodan Zhang, Meijia Zhang

**Affiliations:** The Innovation Centre of Ministry of Education for Development and Diseases, the Second Affiliated Hospital, School of Medicine, South China University of Technology, Guangzhou, China

**Keywords:** Reproductive biology, Apoptosis

## Abstract

Mutations in several translation initiation factors are closely associated with premature ovarian insufficiency (POI). In this study, we demonstrated that the conditional knockout of eukaryotic initiation factor 2 (eIF2) subunits *Eif2s1* and *Eif2s2* in mouse oocytes caused oocyte apoptosis within the early growing follicles. Subsequent research indicated that the depletion of *Eif2s2* in oocytes reduced the levels of mitochondrial fission-related proteins (p-DRP1, FIS1 and MFF) and increased the mRNA and protein levels of the integrated stress response (ISR)-related factors (ASNS, SLC7A1, GRB10 and PSAT1). Consistent with this, the depletion of *Eif2s2* in oocytes resulted in mitochondrial dysfunction characterized by elongated form, aggregated distribution beneath the oocyte membrane, decreased mitochondrial membrane potential and ATP content, and excessive accumulation of reactive oxygen species (ROS). At the same time, the depletion of *Eif2s2* in oocytes led to increased levels of DNA damage response proteins (γH2AX, p-CHK2 and p53) and proapoptotic proteins (BAX and PARP1), as well as decreased the levels of anti-apoptotic protein BCL-xL. Collectively, these findings indicate that the depletion of eIF2 subunits in mouse oocytes leads to oocyte apoptosis within the early growing follicles, attributed to the impaired translation of mitochondrial dynamics regulatory proteins and then the upregulated ROS levels and DNA damage. This study provides new insights into pathogenesis and genetic diagnosis for POI.

## Introduction

Ovarian follicle is the basic functional unit of female reproduction [1]. In mammals, the nonrenewable primordial follicles are formed in the ovary during the perinatal period and determine the female reproductive lifespan [2]. Overactivation of primordial follicles accelerates the depletion of the follicle pool and leads to premature ovarian insufficiency (POI) [3]. During each follicular wave, only a few primordial follicles are activated, and most still maintain a non-growing state with minimal transcription and translation activity to provide a continuous oocyte supply [2, 4]. Activated oocytes undergo a growth stage marked by volume expansion, organelle biogenesis, mRNA production, and protein biosynthesis [5]. Rapid biogenesis of mitochondrion by mitochondrial fission and mitochondrial DNA (mtDNA) replication contributes to the regulation of transcription and translation by providing ATP and intermediate metabolites [6–8]. At the same time, oocyte-derived paracrine factors, such as bone morphogenetic protein 15 (BMP15) and growth differentiation factor 9 (GDF9), support the metabolic activity and proliferation of granulosa cells, which in turn promotes oocyte development by providing pyruvate, amino acids and steroids via transzonal projections (TZPs) and gap junctions [9–11]. The oocyte exhibits transcriptional silencing and gains the ability to resume meiosis when the non-surrounded nucleolus (NSN) develops into the surrounded nucleolus (SN) [5, 12]. Subsequently, oocyte meiotic maturation relies on the translation of maternal RNAs stored in the oocyte cytoplasm [13, 14]. Therefore, the regulation of protein translation in both space and time is essential for oocyte growth and meiotic maturation.

Eukaryotic initiation factor 2 subunits 1, 2 and 3 (eIF2S1, 2 and 3, also known as eIF2α, β and γ) form the multimeric protein complex named eIF2 [15]. During each round of translation, eIF2 forms the ternary complex (TC) with GTP and initiator methionyl-tRNA (Met-tRNAi) [16]. Then, TC (eIF2·GTP·Met-tRNAi) binds to 40S ribosomal subunit, eIF1, eIF1A, eIF3 and eIF5 to form 43S preinitiation complex (PIC) [17]. 43S-PIC is subsequently recruited to mRNA by the eIF4 complex and then scans and searches for an initiation codon in the mRNA 5′ untranslated region (5′ UTR) to initiate translation [18, 19]. Immediately afterward, eIF2·GTP is hydrolyzed by eIF5 to an inactive form, eIF2·GDP, and then eIF2·GDP is released from the mRNA. A fresh cycle of mRNA translation is initiated when eIF2B converts eIF2·GDP into eIF2·GTP [20, 21].

In humans, the mutations of eukaryotic translation initiation factors *EIF2B2*, *EIF2B4*, *EIF2B5* and *EIF4ENIF1* (EIF4E nuclear import factor) are closely associated with POI, but the pathogenic causality and mechanisms remain largely unknown [22–24]. In mice, *Eif4enif1* haploinsufficiency leads to excessive mitochondrial hyperfusion and altered distribution of mitochondrial-associated ribonucleoprotein domains by affecting mitochondrial fission and fusion protein translation, resulting in low fertility [25]. eIF4E family member 1B (*Eif4e1b*) deletion in oocytes resulted in defective embryonic development and infertility via impairing the translation of maternal mRNA [26]. In addition, the deficiency in several translation-related factors can also cause female infertility. For example, damaged DNA-binding protein 1 and cullin-ring finger ligase 4 (CUL4) associated factor 13 (*Dcaf13*) deletion in oocytes stunts chromatin configuration transition by downregulating global protein translation, leading to oocyte development arrest [5]. Double deletion of zygote arrest-1 and 2 (*Zar1/2*) in germ cells causes defects in meiosis resumption and early embryonic development via impairing translational activation of maternal mRNAs [27]. In short, these findings suggest that imperfections in distinct translation regulators can cause various female fertility defects, including POI-like phenotype.

eIF2, containing α, β and γ subunits, is the core complex of translation initiation. In galactosemia model mice, the elevated eIF2α phosphorylation levels and endoplasmic reticulum (ER) stress compromise the survival of the primordial follicle and accelerate ovarian aging [28], although the short-term increase of eIF2α phosphorylation is beneficial for cell survival during stress-induced integrated stress response (ISR) [29]. Our study has found that *Eif2s2* deletion in premeiotic germ cells causes oocyte arrest at the early diplotene stage and apoptosis via impairing homologous recombination and mitochondrial function [30], and *Eif5* depletion in oocytes causes apoptosis through reactive oxygen species (ROS) accumulation and DNA damage pathway [31]. The targeted mutation of *Eif2s3y* prevents spermatogonia differentiation and spermatogenesis, leading to male sterility [32]. Nevertheless, the function of eIF2 in oocyte growth and folliculogenesis remains unclear.

Here, conditional deletion of *Eif2s1* and *Eif2s2* in primordial and primary oocytes uncovered the roles of eIF2α and eIF2β on mouse oocyte growth and folliculogenesis. The results showed that the deletion of *Eif2s1* and *Eif2s2* in mouse oocytes caused oocyte apoptosis in early growing follicles. Correspondingly, these phenotype defects resulted from the impaired translation of mitochondrial dynamics regulatory proteins and then the upregulated ROS levels and DNA damage in oocytes.

## Materials and methods

### Animals

The *Eif2s1*^flox/flox^ and *Eif2s2*^flox/flox^ mice were procured from GemPharmatech Co., Ltd. The *Eif2s1* and *Eif2s2* conditional knockout mice were bred by crossbreeding the *Eif2s1*^flox/flox^ and *Eif2s2*^flox/flox^ females with *Gdf9*-Cre males (hereafter designated *Eif2s1-*GcKO and *Eif2s2-*GcKO) and *Zp3*-Cre males (hereafter designated *Eif2s1-*ZcKO and *Eif2s2-*ZcKO), respectively. Genomic DNA from mice was used to identify the genotype by PCR, and primers for genotyping are shown in Table S1. All animal research was approved by the South China University of Technology’s Institutional Animal Care and Use Committee.

### Morphological examination and follicle counting

The ovaries collected from WT, *Eif2s1* and *Eif2s2* cKO mice at different ages were preserved in 4% paraformaldehyde (P1110, Solarbio) for 12 h. Following this, the ovaries were subjected to dehydration, embedding, and sectioning. Sections of 5 micrometers were then stained, and the count of primordial, primary, early secondary (2 layers of granulosa cells), late secondary (≥3 layers of granulosa cells) and antral follicles was conducted using the method as previously described [26]. All follicle counts were performed by blinded observers.

### Oocyte isolation and in vitro maturation

At postnatal days 21 (PD21), the WT, *Eif2s1* and *Eif2s2* cKO mice were injected with 5 IU equine chorionic gonadotropin (eCG). 48 h after PMSG injection, the oocytes at the germinal vesicle (GV) stage were carefully isolated and collected from cumulus-oocyte complexes (COCs) within the ovaries. The GV oocytes were cultured in an M16 medium covered with mineral oil at 37 °C, and then the rate of oocytes with germinal vesicle breakdown (GVBD) and first polar body (PB1) extrusion was calculated after culturing for 3 and 14 h, respectively. To collect metaphase II (MII)-stage oocytes, the female mice were treated with 5 IU eCG, followed by 5 IU human chorionic gonadotropin (hCG) 48 h later. Finally, the MII oocytes were harvested from COCs in the mouse oviducts 13 h post-hCG injection. For ROS scavenger experiments, we cultured GV oocytes from *Eif2s2*-ZcKO mice without or with 2 mM N-acetylcysteine (NAC. S0077, Beyotime) for 14 h to examine the rate of oocytes with GVBD and PB1.

### Immunofluorescence staining

The GV oocytes collected from WT, *Eif2s1* and *Eif2s2* cKO mice were successively placed in 4% PFA, 0.25% Triton X-100 and 3% BSA to achieve fixation, permeabilization and blocking. After these steps, the oocytes were incubated with the primary antibodies at 4 °C overnight, followed by incubation with fluorescent secondary antibodies at 37 °C for 1 h. Finally, the oocytes were counterstained with 4′, 6-diamidino-2-phenylindole (DAPI) and sealed in an anti-fluorescence quencher. The immunofluorescence staining of ovarian sections was performed following a procedure described previously [33]. Image acquisition of oocytes and ovarian sections was performed using an LSM 800 confocal microscope with the same parameters, and then the fluorescence intensity was measured by ZEN software (Carl Zeiss, Version 3.1). All positive signals were assessed by the blinded observers.

### Transmission electron microscopy

The GV oocytes collected from eCG-primed mice were placed in 2.5% glutaraldehyde for fixation, followed by pre-embedding with agarose. After washing with PBS, the oocytes underwent post-fixation, dehydration, resin-embedding, sectioning and counterstaining following previously described procedures. Finally, image acquisition of oocytes was performed using Transmission electron microscopy (TEM) (FEI, Tecnai G2 Spirit, 120 kV).

### Bromodeoxyuridine (BrdU) incorporation assay

The WT and *Eif2s2*-ZcKO mice were injected with BrdU (100 mg/kg) 4 h before sacrifice. Subsequently, the ovaries were collected to prepare tissue sections, and the sections were subjected to immunofluorescence staining of BrdU. Finally, the percentage of granulosa cells with BrdU-positive signals in primary and secondary follicles of the largest five sections was calculated to assess granulosa cell proliferation.

### TUNEL assay

Apoptosis of ovarian cells was detected by Click-iT™ Plus TUNEL Assay (1982275, Thermo Fisher). Briefly, the dewaxed and rehydrated ovarian sections were first fixed and permeabilized by 4% PFA and proteinase K, respectively, followed by incubation with a TUNEL reaction mixture. After washing with PBS, the sections were counterstained with DAPI, and the image acquisition was performed using an LSM 800 confocal microscope. The TUNEL-positive signals in per section were counted.

### Puromycin incorporation assay

The puromycin incorporation was performed to assess the translation efficiency. Briefly, the WT and *Eif2s2*-ZcKO mice were injected with puromycin (65 mg/kg) 1.5 h before sacrifice. Subsequently, the oocytes were used to extract the total protein for Western blot analysis with an anti-puromycin primary antibody. Relative gray values of anti-puromycin of the total lanes were calculated by ImageJ software, using β-actin as an internal standard.

### RNA isolation and analysis

Total RNA from WT and *Eif2s2*-ZcKO GV oocytes was isolated with a commercial kit (74004, Qiagen), and then the RNA was used to create cDNA by a reverse transcription kit (205311, Qiagen). qRT-PCR was performed to quantify the mRNA levels using a LightCycler 96 instrument (Roche). The relative mRNA level was calculated based on the 2^−ΔΔCt^ method, with ribosomal protein L19 (*Rpl19*) serving as an endogenous control. To perform Smart-seq2 analysis, the GV oocytes were collected from WT and *Eif2s2*-GcKO at PD14, as well as from WT and *Eif2s2*-ZcKO at PD21. All samples were analyzed by the DNBSEQ platform (BGI, China). All primers for qRT-PCR are shown in Table S2.

### Western blot and proteomic analysis

100 GV oocytes collected from each genotype were lysed in a RIPA buffer to extract total protein. Subsequently, the protein samples underwent separation with an SDS-PAGE gel and were transferred to a PVDF membrane. After blocking with 5% skim milk, the membranes were incubated with primary antibodies at 4 °C overnight, followed by incubation with appropriate secondary antibodies (1:5000, ZSGB-BIO). Finally, all membranes were exposed to Immobilon Western Chemiluminescent HRP substrate (Millipore), and bands were detected using a Tanon 5200 imaging system. Relative gray values of protein bands were calculated by ImageJ software, using β-actin as an internal standard. All primary antibodies are detailed in Table S3. For proteomic analysis, total protein was extracted from 3000 oocytes of each genotype and then analyzed by PTM-biolab (China).

### Active mitochondrial staining

GV oocytes were incubated with 100 nM MitoTracker Deep Red FM (C1032, Beyotime) for 30 min at 37 °C, followed by washing with preheated M2 medium. Subsequently, these oocytes were counterstained with Hoechst 33342 and submitted to a confocal microscope for image acquisition.

### Measurement of mitochondrial membrane potential

GV oocytes were incubated with JC1 for 30 min at 37 °C (C2003S, Beyotime), followed by washing with preheated M2 medium. Finally, the image acquisition of living oocytes was performed using a confocal microscope, and mitochondrial membrane potential (MMP) is indicated by a ratio of red fluorescence intensity (J-aggregates) to green fluorescence intensity (J-monomers).

### Detection of reactive oxygen species (ROS) and mitochondrial superoxide (MitoSOX)

The total ROS levels and MitoSOX of GV oocytes were detected by commercial kits (S0033S, Beyotime; RM02822, Abclonal). Briefly, the oocytes were incubated in a serum-free medium with 10 μM DCFH-DA for 30 min at 37 °C for ROS detection. The oocytes were cultured in Hank’s balanced salt solution with 5 μM Mitochondrial Superoxide indicator for 30 min at 37 °C for MitoSOX detection. Finally, all oocytes were submitted to a confocal microscope for image acquisition and analysis.

### Detection of ATP content

The ATP content of oocytes from WT and *Eif2s2*-ZcKO mice was detected with a commercial kit (S0027, Beyotime). Specifically, 15 GV oocytes of each genotype were transferred into a lysis buffer and incubated for 10 min on ice. After centrifugation, 10 μL of supernatant and gradient concentration of the standards were submitted to a microplate reader to measure the optical density value. Finally, the ATP content was calculated based on the standard curve generated with the standards.

### Annexin V staining

The oocyte apoptosis was assessed using an Annexin V-mCherry detection kit (C1069S, Beyotime) according to the provided protocol. Specifically, the GV oocytes from WT and *Eif2s2*-ZcKO mice were washed by preheated M2 medium, and then were incubated for 20 min in the dark with a working solution containing 195 μL binding buffer and 5 μL Annexin V-mCherry. After washing three times with M2 medium, the oocytes were observed under the confocal microscope.

### Mitochondrial DNA copy number measurement

The quantification of mitochondrial DNA (mtDNA) copy number was performed as previously described [34]. Total genomic DNA from WT and *Eif2s2*-ZcKO oocytes was extracted using the TIANamp Genomic DNA kit (TIANGEN, Beijing, China). Selected the NADH dehydrogenase subunit 1 and Hexokinase 2 (*HK2*) gene (Table S4) to evaluate the relative copy number of mtDNA and nuclear DNA (nDNA) for our assay. qRT-PCR was performed to measure the ratio of mtDNA to nDNA, thereby quantifying the mtDNA copy number.

### Ovary tissue culture

Four weeks *Eif2s2*-ZcKO mice were euthanized, and the ovaries were removed and cut into four equal parts in sterile phosphate-buffered saline (PBS). Then, the ovarian fragments were placed on a Millipore insert (Millipore, Billerica, MA, USA) in a six-well culture plate (NEST, Beijing, China). Each well contained 3 mL of Dulbecco’s modified Eagle’s medium/Ham’s F12, and its components have been reported previously [35]. 100 ng/mL murine recombinant anti-Müllerian hormone (AMH, HY-P72077, MedChemExpress, China) was added as needed. The ovaries were cultured at 37 °C and 5% CO_2_, and the medium was changed every 2 days. After 4 days of culture, the fragments were harvested for immunofluorescence staining and follicle counting.

### Statistical analysis

The experimental data are expressed as the mean ± standard deviation (SD), and at least three biological replicates were conducted for each experiment. For significance analysis and data visualization, the Student’s *t* test and GraphPad Prism 8.3 were used in the present study.

## Results

### Oocyte-expressed eIF2α and eIF2β are indispensable for follicle development and female fertility

**Fig. 1:**
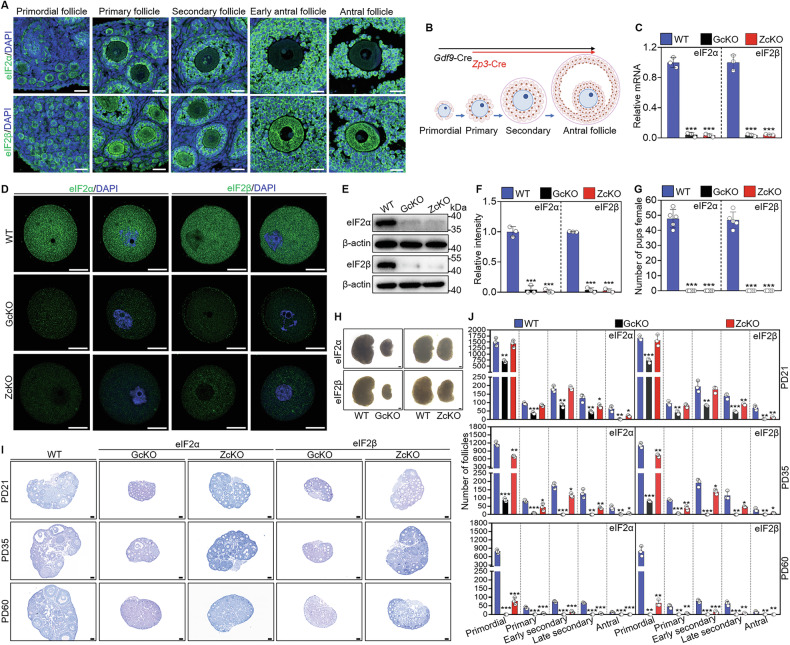
Oocyte-specific deletion of *Eif2s1* and *Eif2s2* causes mouse follicle development defects and infertility. **A** Immunofluorescence images of ovarian sections stained with anti-eIF2α and eIF2β antibodies (green) and DAPI (blue) for primordial, primary, secondary, and antral follicular stages. **B** Schematic representation showing oocyte-specific knockout of *Eif2s1* and *Eif2s2* at primordial and primary follicle stages by *Gdf9*-Cre (GcKO) and *Zp3*-Cre (ZcKO), respectively. **C** qRT-PCR analysis of mRNA levels of *Eif2s1* and *Eif2s2* in WT, GcKO and ZcKO oocytes. **D** Immunofluorescence images of eIF2α and eIF2β in WT, GcKO and ZcKO oocytes at postnatal days 21 (PD21). **E**, **F** Western blot results of eIF2α and eIF2β protein levels in WT, GcKO and ZcKO oocytes. **G** Cumulative numbers of pups per female among the WT, GcKO and ZcKO mice (*n* = 5 females for each genotype) during the 40-week fertility assessment. **H** Representative images of ovaries isolated from WT, GcKO and ZcKO female mice at PD21. **I** PAS staining showing ovarian histology of WT, GcKO and ZcKO female mice at the indicated ages. **J** Quantification of the number of follicles at different developmental stages, *n* = 3 females for each genotype. In each experiment, *n* ≥ 3 biological replicates. Bars indicate the mean ± SD. A two-sided Student’s t-test was used to determine *P* values. (*P < 0.05, **P < 0.01 and ***P < 0.001). Scale bar: 25 μm (**A**, **D**) and 100 μm (**I**, **H**).

Immunofluorescence staining showed that eIF2α and eIF2β were expressed in granulosa cells and oocytes throughout follicular development (Fig. 1A). Therefore, we generated *Eif2s1*- and *Eif2s2*-GcKO mice, as well as *Eif2s1*- and *Eif2s2-*ZcKO mice, to investigate the role of eIF2α and eIF2β in oocyte development (Fig. 1B and Supplementary Fig. 1A). The knockout efficiency of *Eif2s1* and *Eif2s2* was validated by qRT-PCR, immunofluorescence and Western blot. The results also showed that a trace amount of residual eIF2α and eIF2β persisted in the oocytes of *Eif2s1* and *Eif2s2* cKO mice, respectively (Fig. 1C–F and Supplementary Fig. 1B).

Fertility testing showed that the depletion of *Eif2s1* and *Eif2s2* both resulted in complete female sterility (Fig. 1G). Morphological observation showed that the ovarian size was significantly smaller in *Eif2s1* and *Eif2s2* cKO mice than in WT mice at PD21 (Fig. 1H and Supplementary Fig. 1C). PAS staining and follicle counting results showed that the number of primary, early secondary and late secondary follicles was significantly decreased in *Eif2s1*- and *Eif2s2*-GcKO ovaries at PD14 (Supplementary Fig. 1D). At PD21, the number of all stage follicles was significantly decreased in *Eif2s1*- and *Eif2s2*-GcKO mice ovaries (Fig. 1I, J). The number of late secondary and antral follicles, not the number of primordial, primary and early secondary follicles, in *Eif2s1*- and *Eif2s2*-ZcKO mice ovaries was significantly decreased at PD21 compared with the corresponding WT mice ovaries (Fig. 1I, J). At PD35, the number of all stage follicles was significantly decreased in *Eif2s1*- and *Eif2s2*-ZcKO mice ovaries (Fig. 1I, J). It was worth noting that the primordial follicles with eIF2α and eIF2β expression also declined concurrently with diminished growing follicles in *Eif2s1*- and *Eif2s2*-ZcKO mice ovaries (Fig. 1J). In contrast to the corresponding WT mice, the mature follicles were not observed in *Eif2s1*- and *Eif2s2*-GcKO ovaries at PD21 and in *Eif2s1*- and *Eif2s2*-ZcKO ovaries at PD35 (Fig. 1I, J), suggesting that GcKO mice exhibit an early onset of premature ovarian failure at PD21, while ZcKO mice suffer from it at PD35. Furthermore, all follicles were exhausted in *Eif2s1* and *Eif2s2* cKO mice at PD180 (Supplementary Fig. 1E). These findings suggest that the depletion of *Eif2s1* and *Eif2s2* in oocytes results in follicle development disabilities, ultimately leading to female infertility.

### Depletion of *Eif2s2* in oocytes causes defects in oocyte meiotic maturation

**Fig. 2:**
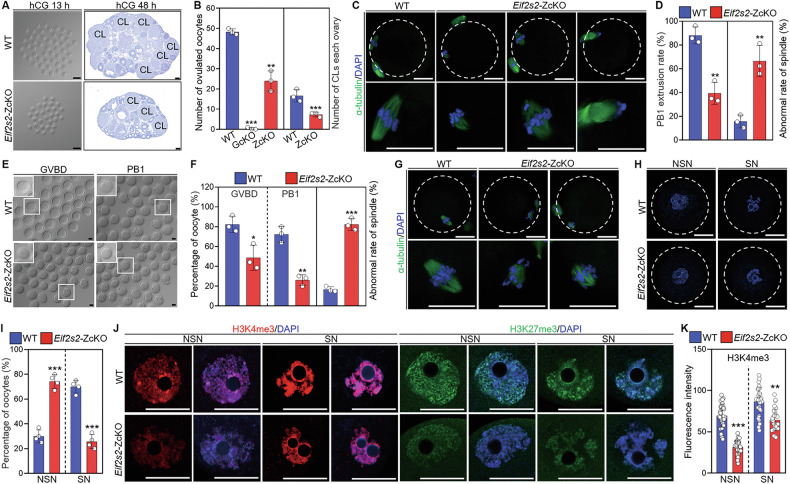
Depletion of *Eif2s2* in oocytes impairs oocyte maturation. **A** Representative image of oocytes ovulated and corpus luteum 13 h and 48 h after human chorionic gonadotropin (hCG) treatment. **B** The number of ovulatory oocytes per female mouse and corpora lutea in each ovary, *n* = 3 females each genotype. **C** Immunofluorescence staining of α-tubulin showing spindle assembly in oocytes ovulated by WT and *Eif2s2*-ZcKO mice. **D** The percentage of first polar body (PB1) extruded (WT: *n* = 165, *Eif2s2*-ZcKO: n = 105) and abnormal spindle assembly in superovulated oocytes of WT and *Eif2s2*-ZcKO mice. **E**–**G** Representative images and the percentage of germinal vesicle breakdown (GVBD), PB1 extrusion and abnormal spindle assembly in WT and *Eif2s2*-ZcKO oocytes cultured in vitro. *n* = 3, and 61-94 oocytes were analyzed in each repetition. **H** DAPI staining of the oocytes with non-surrounded nucleolus (NSN) and surrounded nucleolus (SN) chromatin configurations in WT and *Eif2s2*-ZcKO oocytes. **I** The percentage of NSN and SN type oocytes isolated from WT (*n* = 244 oocytes) and *Eif2s2*-ZcKO (*n* = 168 oocytes) mice. **J** Immunofluorescence staining of H3K4me3 and H3K27me3 in WT and *Eif2s2*-ZcKO oocytes. **K** Quantification of H3K4me3 fluorescence intensity (*n* = 30 oocytes). In each experiment, *n* ≥ 3 biological replicates. Bars indicate the mean ± SD. A two-sided Student’s t-test was used to determine *P* values. (*P < 0.05, **P < 0.01, and ***P < 0.001). Scale bar: 100 μm (**A**) and 25 μm (**C**, **E**, **G**, **H**, **J**).

The *Eif2s2* cKO mice were selected to assess the effects of oocyte developmental competence since the *Eif2s1* and *Eif2s2* cKO mice have similar reproductive phenotypes. The results of superovulation showed that ovulation number was significantly reduced in *Eif2s2*-ZcKO mice versus WT mice (24 vs 48; Fig. 2A, B), while the *Eif2s2*-GcKO mice failed to ovulate (Fig. 2B). Consistently, *Eif2s2*-ZcKO ovaries showed a significant reduction in corpora lutea (CLs) 48 h post-hCG (Fig. 2A, B). Further analyses showed that only 39.4% of *Eif2s2*-ZcKO mice ovulated oocytes PB1 (Fig. 2D), and these oocytes with PB1 showed an increased percentage of abnormal spindles (66.52% vs 15.7%) contrasted to the WT oocytes (Fig. 2C, D). Similarly, these defects were recapitulated when the fully grown oocytes (FGOs) from *Eif2s2*-ZcKO mice underwent maturation in vitro (Fig. 2E–G). Additionally, samples of FGOs obtained from *Eif2s2*-ZcKO and WT mice underwent evaluation. Analysis of chromatin configuration showed that 70% of the oocytes were SN in WT mice, while only 25.75% of the oocytes were SN in *Eif2s2*-ZcKO mice (Fig. 2H, I), suggesting that the nuclear development of oocytes from *Eif2s2*-ZcKO mice is still arrested at the NSN stage. The buildup of H3K4 trimethylation (H3K4me3) is indispensable for the chromatin configuration transition from NSN to SN [36]. IF analysis revealed markedly reduced H3K4me3 and H3K27me3 signal intensity in *Eif2s2*-ZcKO NSN and SN oocytes relative to WT oocytes (Fig. 2J, K and Supplementary Fig. 2A–C), suggesting that nuclear development defects are present in these oocytes as well. These results indicate that *Eif2s2* deletion in oocytes leads to disruptions in nuclear maturation and the progression of meiosis.

### Depletion of *Eif2s2* in oocytes causes injury in oocyte-granulosa cell communication

**Fig. 3:**
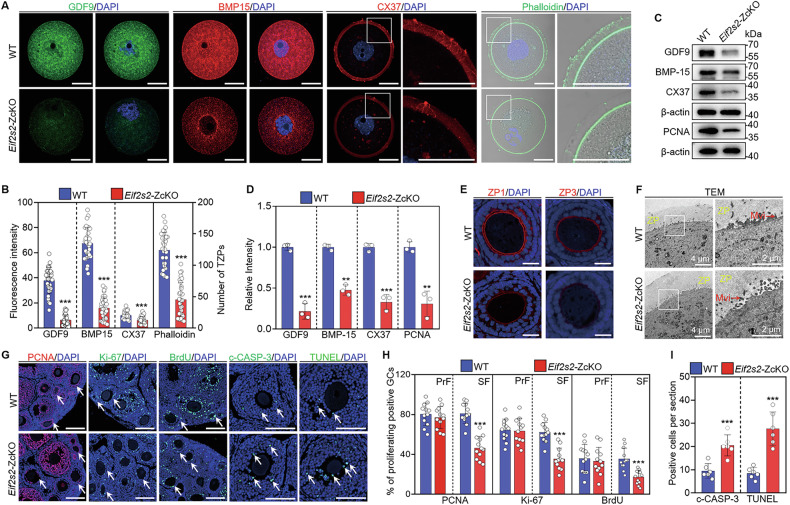
Depletion of *Eif2s2* in oocytes impairs granulosa cell proliferation and induces apoptosis. **A** Immunofluorescence staining of GDF9, BMP15, CX37 and Phalloidin in WT and *Eif2s2*-ZcKO oocytes. **B** Quantification of fluorescence intensity and transzonal projections (TZPs) number in WT and *Eif2s2*-ZcKO oocytes (*n* = 30). **C**, **D** Western blot analysis of GDF9, BMP15 and CX37 levels in WT and *Eif2s2*-ZcKO oocytes, and PCNA levels in WT and *Eif2s2*-ZcKO granulosa cells. **E** Immunofluorescence staining of ZP1 and ZP3 in WT and *Eif2s2*-ZcKO oocytes. **F** Transmission electron microscopic (TEM) images of WT and *Eif2s2*-ZcKO oocytes. ZP, zona pellucida. Mvi, microvillus. **G** Immunofluorescence staining of Ki-67, PCNA, BrdU, TUNEL, and c-CASP-3 in WT and *Eif2s2*-ZcKO ovaries at PD21. **H** The percentage of granulosa cells with Ki-67-, PCNA-, and BrdU-positive signals in the primary follicles and secondary follicles. PrF, primary follicle. SF, secondary follicle. **I** Quantification of granulosa cells with TUNEL- and c-CASP-3-positive signals in WT and *Eif2s2*-ZcKO ovaries at PD21. In each experiment, *n* ≥ 3 biological replicates. Bars indicate the mean ± SD. A two-sided Student’s *t* test was used to determine *P* values. (**P < 0.01 and ***P < 0.001). Scale bar: 25 μm (**A**, **E**) and 100 μm (**G**).

Follicle and oocyte development defects are closely associated with impaired bidirectional oocyte-granulosa cell communication [37]. Immunofluorescence staining and Western blot analysis showed the protein levels of BMP15, CX37 and GDF9 in *Eif2s2*-ZcKO oocytes were significantly decreased compared to the WT oocytes (Fig. 3A–D). Moreover, the phalloidin staining showed a disorganized distribution and a decreased number of F-actin bundles in the ZP area of *Eif2s2*-ZcKO oocytes compared to the WT oocytes (Fig. 3A, B). Immunofluorescence staining of ZP1 and ZP3 also revealed thinner ZPs in *Eif2s2*-ZcKO oocytes (Fig. 3E and Supplementary Fig. 3A). Interestingly, microvilli in *Eif2s2*-ZcKO oocytes appeared degenerate under TEM examination, distinctly differing from WT oocytes’ long and unimpaired microvilli extending into the perivitelline space (Fig. 3F). The quantitative analysis results showed that the length of microvilli in *Eif2s2*-ZcKO oocytes was significantly decreased compared with that in WT oocytes (Supplementary Fig. 3E).

To further reveal the causes of follicle development defects, Western blot, immunofluorescence staining and TUNEL were performed to assess the proliferation and apoptosis of granulosa cells. Experimental results showed a significant decrease in granulosa cells expressing PCNA-, Ki-67-, and BrdU-positive signals (Fig. 3G, H and Supplementary Fig. 3B), alongside a significant increase in granulosa cells displaying TUNEL- and cleaved Caspase-3 (c-CASP-3)-positive signals in the secondary follicles of *Eif2s2*-ZcKO mice compared to the WT mice (Fig. 3G, I and Supplementary Fig. 3C). Continuously, the expression of PCNA and/or Ki-67 mRNA and proteins was markedly reduced in *Eif2s2*-ZcKO granulosa cells (Fig. 3C, D and Supplementary Fig. 3D). In short, the depletion of *Eif2s2* in oocytes leads to granulosa cell proliferation defects and apoptosis, possibly due to the oocyte-granulosa cell communication defects and decreased expression of oocyte-secreted factors.

### Depletion of *Eif2s2* in oocytes disrupts the protein expression profile

**Fig. 4:**
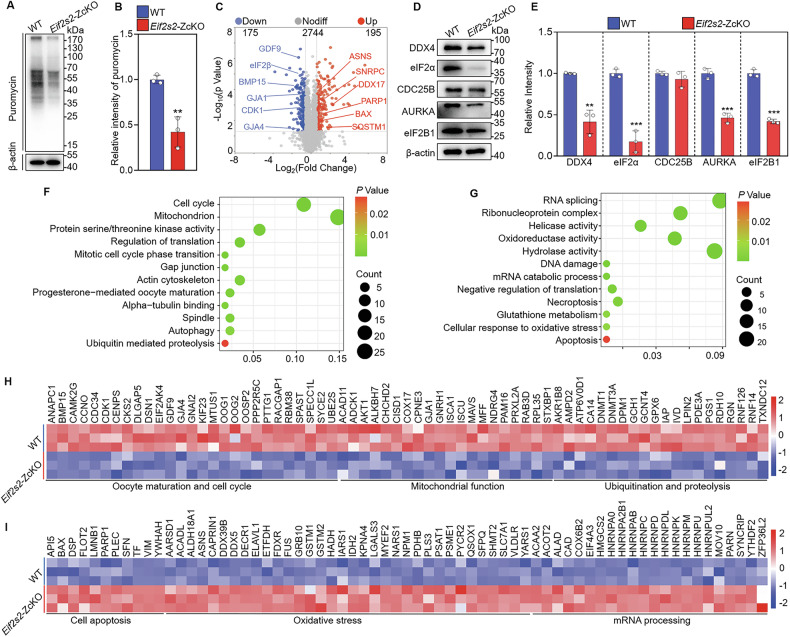
Depletion of *Eif2s2* in oocytes inhibits global protein translation. **A**, **B** Western blot analysis of puromycin incorporation in WT and *Eif2s2*-ZcKO oocytes. **C** Volcano plot illustrating the differentially expressed proteins in WT and *Eif2s2*-ZcKO oocytes. **D**, **E** Western blot analysis of DDX4, eIF2B1, eIF2α, AURKA, CDC25B and β-actin expression in WT and *Eif2s2*-ZcKO oocytes. **F**, **G** Bubble chart illustrating the enriched GO terms associated with the significantly downregulated and upregulated proteins in *Eif2s2*-ZcKO oocytes. **H**, **I** Heatmaps illustrating downregulation and upregulation between WT and *Eif2s2*-ZcKO oocytes in the expression of a group of proteins involved in various processes. In each experiment, *n* ≥ 3 biological replicates. Bars indicate the mean ± SD. A two-sided Student’s *t* test was used to determine *P* values. (**P < 0.01 and ***P < 0.001).

eIF2β is essential for regulating translation initiation by forming the TC. Therefore, the translational efficiency was first detected using the puromycin incorporation method. Western blot analysis showed that the anti-puromycin signal was markedly reduced in *Eif2s2*-ZcKO oocytes compared to the WT oocytes (Fig. 4A, B), implying a reduction in the creation of novel proteins. Further proteomic analysis showed the detection of 3114 distinct proteins (Fig. 4C). Compared to the WT oocytes, 175 proteins were decreased and 195 proteins were increased by > 1.5-foldchange in *Eif2s2*-ZcKO oocytes (Fig. 4C). The representative proteins (DDX4, eIF2B1, eIF2α, CDC25B and AURKA) selected from proteomic data were validated by Western blot (Fig. 4D, E). Gene Ontology (GO) analysis of the downregulated proteins revealed processes such as oocyte maturation and cell cycle, mitochondrial function, gap junction, ubiquitination and proteolysis (Fig. 4F, H). The significantly decreased proteins COX17 (for electron transport chain assembly) and MFF (for mitochondrial fission) (Fig. 4H) could cause mitochondrial dysfunction in *Eif2s2*-ZcKO oocytes. In addition, the decreased BMP15, CX37 and GDF9 (Fig. 4H) could cause the defects in cholesterol biosynthesis, tricarboxylic acid (TCA) cycle and glycolysis of granulosa cells. The upregulated proteins revealed that the processes such as mRNA processing, DNA damage, oxidative stress and cell apoptosis (Fig. 4G). The increased BAX, PARP1 and SFN in *Eif2s2*-ZcKO oocytes (Fig. 4I) could trigger cell apoptosis. Thus, the depletion of *Eif2s2* in oocytes causes insufficient expression of proteins indispensable for oocyte meiotic maturation and mitochondrial function.

### Mitochondrial dysfunction in oocytes of *Eif2s2*-depletion mice

**Fig. 5:**
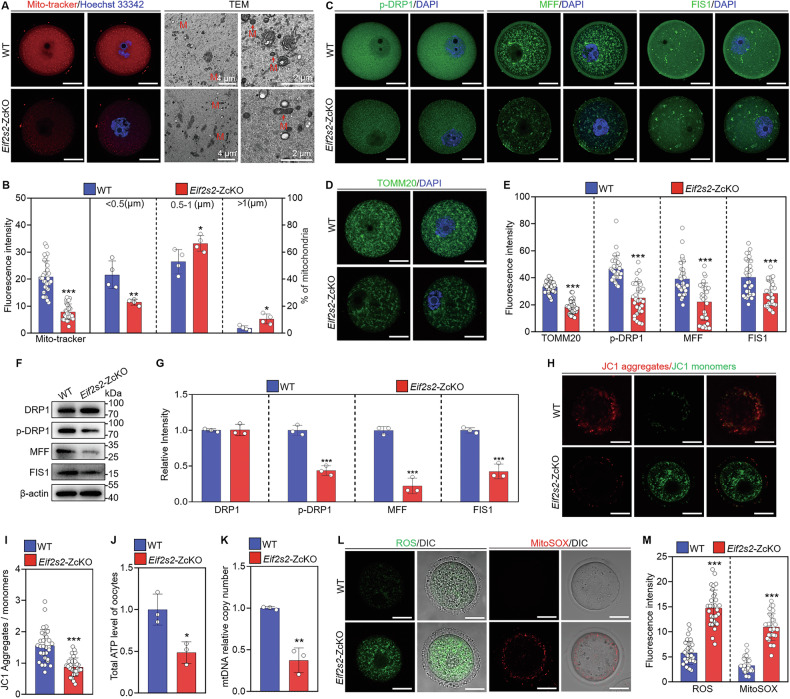
Depletion of *Eif2s2* in oocytes impairs mitochondrial fission and function. **A** Representative images of MitoTracker staining and TEM in WT and *Eif2s2*-ZcKO oocytes. Mitochondrion (M). **B** Quantification of fluorescence intensity of MitoTracker (*n* = 30) and the percentage of mitochondrial length distribution (*n* = 4) in WT and *Eif2s2*-ZcKO oocytes. **C**–**E** Immunofluorescence staining of p-DRP1, FIS1, MFF and TOMM20 and quantification of fluorescence intensity in WT and *Eif2s2*-ZcKO oocytes, *n* = 30 oocytes in each group. **F**, **G** Western blot analysis of DRP1, p-DRP1, FIS1, and MFF expression in WT and *Eif2s2*-ZcKO oocytes. **H** Representative images of mitochondrial membrane potential assessed by JC-1 staining. **I** Histogram showing the JC-1 red/green fluorescence ratio in WT and *Eif2s2*-ZcKO oocytes, *n* = 30 oocytes in each group. **J** Quantitative analysis of the relative ATP levels in WT and *Eif2s2*-ZcKO oocytes, *n* = 45 oocytes in each group. **K** Measurement of the mitochondrial DNA (mtDNA) copy number in WT and *Eif2s2*-ZcKO oocytes, *n* = 100 oocytes in each group. **L** Representative images of reactive oxygen species (ROS) and mitochondrial superoxide (MitoSOX) detected by DCFH staining and MitoSOX staining in WT and *Eif2s2*-ZcKO oocytes. **M** Quantification of fluorescence intensity of ROS and MitoSOX in WT and *Eif2s2*-ZcKO oocytes, *n* = 30 oocytes in each group. In each experiment, *n* ≥ 3 biological replicates. Bars indicate the mean ± SD. A two-sided Student’s *t* test was used to determine *P* values. (*P < 0.05, **P < 0.01, and ***P < 0.001). Scale bar: 25 μm.

The enrichment of an oxidative stress term and the decreased proteins associated with mitochondrial function suggest mitochondrial dysfunction. The mitochondria showed a discrete and even distribution in WT oocyte ooplasm, whereas they clustered below the cell membrane in *Eif2s2*-ZcKO oocytes (Fig. 5A). In addition, the percentage of the elongated mitochondria were significantly increased in *Eif2s2*-ZcKO mice compared to the WT mice (Fig. 5B). Furthermore, the fluorescence signal and protein levels of TOMM20 (a marker for the mitochondrial outer membrane) and mitochondrial fission-related proteins (FIS1, MFF and p-DRP1) were notably decreased in *Eif2s2*-ZcKO oocytes compared to the WT oocytes (Fig. 5C–G). Mitochondrial function analysis showed that the MMP (JC1 aggregates/JC1 monomers), ATP levels, and mtDNA copy numbers were notably decreased (Fig. 5H–K), while the ROS and MitoSOX were notably accumulated in *Eif2s2*-ZcKO oocytes compared to the WT oocytes (Fig. 5L, M). These results indicate that the depletion of *Eif2s2* in oocytes results in mitochondrial dysfunction by impairing the expression of proteins essential for mitochondrial fission.

### Depletion of *Eif2s2* in oocytes causes transcription dysregulation

**Fig. 6:**
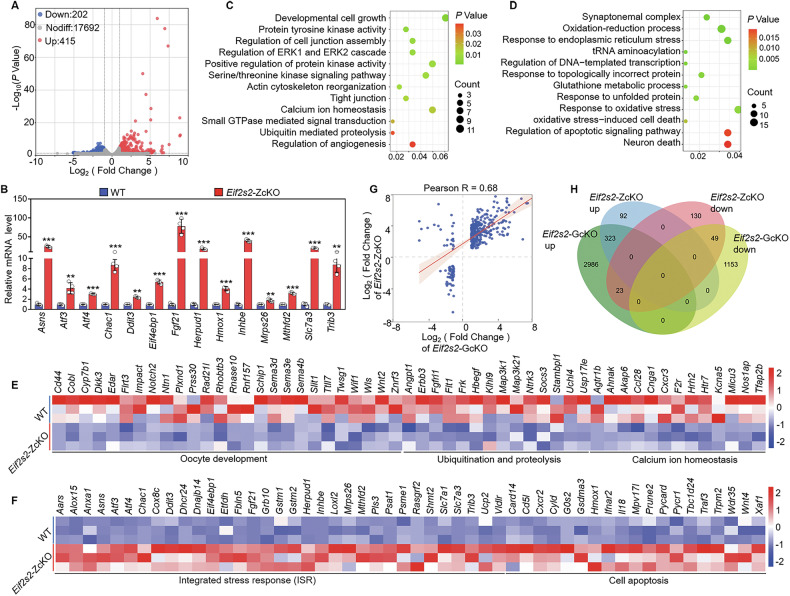
Depletion of *Eif2s2* in oocytes impairs the integrity of the transcriptome. **A** Volcano plot illustrating the differentially expressed transcripts in WT and *Eif2s2*-ZcKO oocytes. **B** qRT-PCR validating the changes in the representative transcripts selected from RNA-seq data. **C**, **D** Bubble chart illustrating the enriched GO terms associated with the significantly downregulated and upregulated transcripts in *Eif2s2*-ZcKO oocytes. **E**, **F** Heatmaps illustrating downregulation and upregulation between WT and *Eif2s2*-ZcKO oocytes in the expression of a group of transcripts involved in various processes. **G** Scatter Plot illustrating the correlation of differentially expressed transcripts between *Eif2s2*-GcKO and *Eif2s2*-ZcKO. **H** Venn diagram illustrating the relationship of up- and down-regulated transcripts identified by RNA-seq in *Eif2s2*-GcKO and *Eif2s2*-ZcKO oocytes. In each experiment, *n* ≥ 3 biological replicates. Bars indicate the mean ± SD. A two-sided Student’s *t* test was used to determine *P* values. (**P < 0.01 and ***P < 0.001).

To confirm whether *Eif2s2*-deletion-induced proteomics shifts resulted from translational rather than transcriptional control, RNA-seq analysis was performed on WT and *Eif2s2*-ZcKO oocytes. Compared to the WT oocytes, 202 transcripts were decreased, and 415 transcripts were increased by > 2-fold change in *Eif2s2*-ZcKO oocytes (Fig. 6A). The representative transcripts underwent qRT-PCR confirmation (Fig. 6B). GO analysis of downregulated transcripts revealed the processes such as oocyte development, ubiquitination and proteolysis and calcium ion homeostasis (Fig. 6C, E). The upregulated transcripts revealed processes such as integrated stress response, apoptosis and response to unfolded protein (Fig. 6D, F). Furthermore, the differentially expressed transcripts in *Eif2s2*-ZcKO oocytes showed a significant positive correlation (*R* = 0.68) with those in *Eif2s2*-GcKO oocytes (Fig. 6G, H and Supplementary Fig. 4A–E). Thus, the *Eif2s2* deletion in the oocytes results in translation inhibition, which in turn may result in transcriptional dysregulation.

**Fig. 7:**
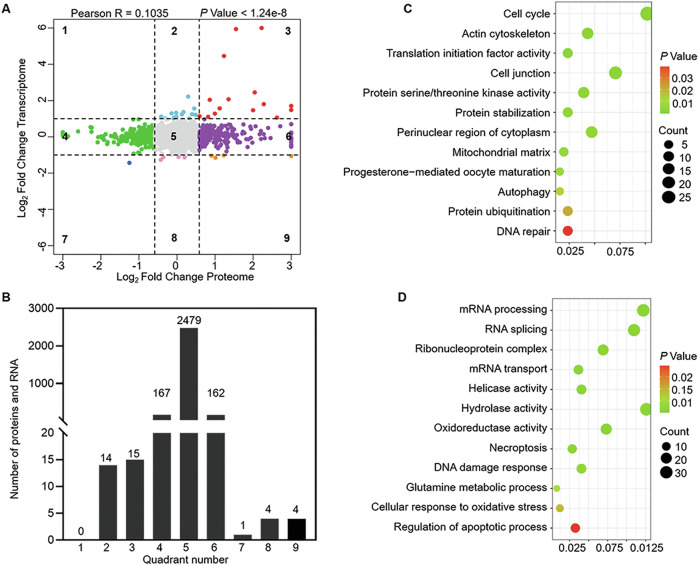
The integration analysis between transcriptome and proteome in *Eif2s2*-ZcKO oocytes. **A** Scatter plot of nine-quadrant associate analysis of transcripts and proteins in WT and *Eif2s2*-ZcKO oocytes. The dashed line on the abscissa represents the FC threshold of transcripts (FC ≥ 2.0), and the dashed line on the ordinate represents the FC threshold of proteins (FC ≥ 1.5). **B** Number of transcripts and proteins enriched in nine quadrants. **C**, **D** Bubble chart illustrating the enriched GO terms associated with proteins in quadrants 1, 2 and 4 and quadrants 6, 8 and 9.

Subsequently, combined transcriptome and proteome analysis was performed through a nine-quadrant diagram (Fig. 7A). Figure 7B illustrates the count of transcripts and proteins in each quadrant. In detail, 2479 proteins in quadrant five demonstrated no alterations in either transcript or protein levels. 14 proteins in the second quadrant and 4 proteins in the eighth quadrant showed a differential expression at transcript levels, but not at protein levels. 167 proteins in the fourth quadrant and 162 proteins in the sixth quadrant showed a differential expression at protein levels, but not at transcript levels. 15 proteins in the third quadrant and 1 protein in the seventh quadrant showed the same trend at the transcript and protein levels. On the contrary, only 4 proteins in the ninth quadrant showed an opposite trend at the transcript and protein levels. Furthermore, the differentially expressed transcripts and proteins within *Eif2s2*-ZcKO oocytes showed no correlation (*R* = 0.1035), similar to the results in oocytes with *Eif4e1b* deletion [26]. GO analysis results of proteins in the first, second and fourth quadrant (translation upregulation), as well as proteins in the sixth, eighth and ninth (translation downregulation) were shown in Fig. 7C, D. Noteworthy, in addition to the activation of ISR marker genes (such as *Atf4*, *Ddit3*, *Chac1*, *Herpud1*, *Trib3*, and *Fgf21*) at the transcriptional level, integrated analysis also showed a significant increase in ISR-related factors (such as ASNS, SLC7A1, GRB10 and GSTM1/2) in *Eif2s2*-ZcKO oocytes at both protein and transcriptional levels (Supplementary Fig. 5). These findings further indicate that depletion of *Eif2s2* in oocytes induces ISR activation.

### Depletion of *Eif2s2* in oocytes triggers DNA damage and oocyte apoptosis

**Fig. 8:**
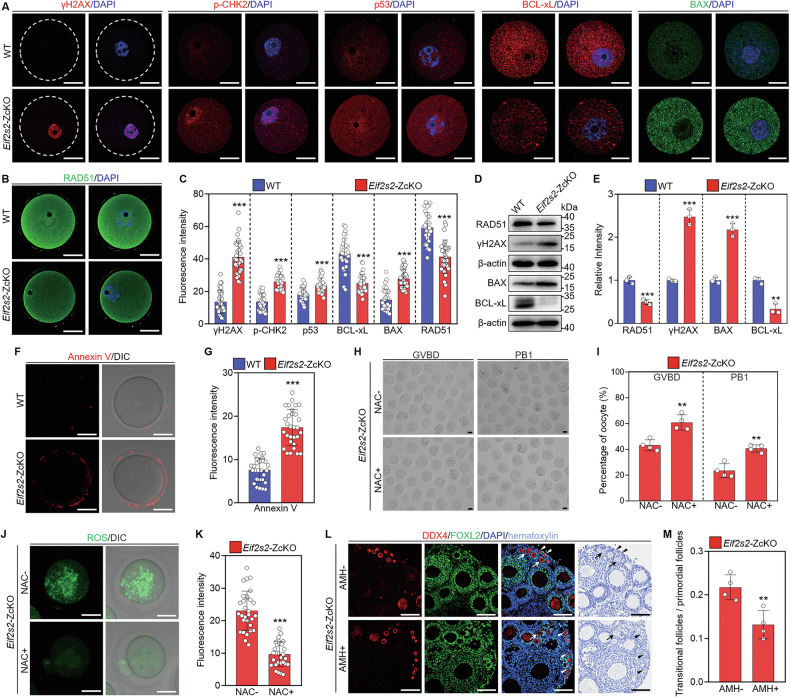
Depletion of *Eif2s2* in oocytes induces DNA damage and oocyte apoptosis. **A** Immunofluorescence staining of γH2AX, p-CHK2, p53, BAX and BCL-xL in WT and *Eif2s2*-ZcKO oocytes. **B** Immunofluorescence staining of RAD51 in WT and *Eif2s2*-ZcKO oocytes. **C** Quantification of fluorescence intensity of γH2AX, p-CHK2, p53, BAX, BCL-xL and RAD51, *n* = 30 oocytes in each group. **D**, **E** Western blot analysis of γH2AX, RAD51, BAX and BCL-xL expression in WT and ZcKO oocytes. **F** Representative images of Annexin V staining in WT and *Eif2s2*-ZcKO oocytes. **G** Quantification of fluorescence intensity of Annexin V (*n* = 30 oocytes). Representative images (**H**) and the percentage (**I**) of oocytes with GVBD and PB1 in *Eif2s2*-ZcKO mice. The oocytes were cultured for 14 h without (NAC-) or with NAC (NAC + ) treatment. *n* = 4, and 52-95 oocytes were analyzed in each repetition. NAC N-acetylcysteine. **J** Representative images of ROS detected by DCFH staining in *Eif2s2*-ZcKO oocytes without or with NAC treatment. **K** Quantification of ROS fluorescence intensity in the oocytes (*n* = 30). **L** Representative images of DDX4, FOXL2 and hematoxylin staining of ovarian sections from *Eif2s2*-ZcKO mice with or without anti-Müllerian hormone (AMH) treatment. **M** Histogram showing the ratio of transitional follicles to primordial follicles (*n* = 4). In each experiment, *n* ≥ three biological replicates. Bars indicate the mean ± SD. A two-sided Student’s *t* test was used to determine *P* values. (**P < 0.01 and ***P < 0.001). Scale bar: 25 μm (**A**, **B**, **F**, **H**, **J**) and 100 μm (**L**).

Excessive accumulated ROS triggers DNA damage and then activates the apoptotic pathway in tubular cells [38]. Immunofluorescence staining and Western blot results showed a significant increase in fluorescence intensity and protein levels of phosphorylated H2AX (γH2AX) (Fig. 8A, C–E), and a significant decrease in the levels of DNA repair protein RAD51 was detected in *Eif2s2*-ZcKO oocytes (Fig. 8B–E). Coherently, the fluorescence intensity of DNA damage response mediators, phosphorylated CHK2 (p-CHK2) and p53, exhibited a notable enhancement in *Eif2s2*-ZcKO oocytes compared to the WT oocytes (Fig. 8A, C). This suggests the increased DNA damage and the decreased DNA damage repair in *Eif2s2*-ZcKO oocytes. As expected, the increased fluorescence intensity and levels of proapoptotic protein BAX, along with decreased fluorescence intensity and levels of anti-apoptotic protein BCL-xL, were detected in *Eif2s2*-ZcKO oocytes (Fig. 8A, C–E). A significantly increased fluorescence intensity of Annexin V is detected in *Eif2s2*-ZcKO oocytes (Fig. 8F, G), indicating oocyte apoptosis. Moreover, the antioxidant NAC significantly decreased ROS levels and Annexin V fluorescence intensity, and increased the rate of *Eif2s2*-ZcKO oocytes with GVBD and PB1 (Fig. 8H–K, and Supplementary Fig. 6A, B), suggesting that ROS accumulation is a causative factor in triggering oocyte apoptosis. The findings suggest that the depletion of *Eif2s2* in oocytes triggers ROS-induced DNA damage and ultimately leads to oocyte apoptosis.

AMH is one such growth factor that is produced by granulosa cells of growing follicles and plays a crucial role in protecting ovarian reserve by preventing the transition of primary follicles from primordial follicles [39, 40]. Thus, we aimed to investigate whether AMH treatment can rescue primordial follicle loss caused by *Eif2s2* depletion-induced apoptosis of growing follicles. *Eif2s2*-ZcKO mouse ovarian tissues were cultured without or with AMH for 4 days, and the results of follicle counting showed that AMH treatment significantly decreased the ratio of transitional follicles to primordial follicles compared with the control (Fig. 8L, M). This suggests that AMH treatment ameliorates primordial follicle loss in *Eif2s2*-ZcKO mice.

## Discussion

The mutations in translation initiation-related factors are closely associated with POI. Our results suggest that targeted deletion of *Eif2s1* and *Eif2s2* causes the arrest of early growing follicle development. The mechanism involves impaired mitochondrial fission protein translation, increased ROS, and activated apoptotic pathway induced by DNA damage (Supplementary Fig. 7).

*Eif2s2* deletion in oocytes downregulated the translation of mitochondrial fission proteins, impaired mitochondrial function, and increased the levels of ROS, resulting in oocyte apoptosis. And the deletion of the mitochondrial fission protein genes *Mff* and *Drp1* induces oocyte mitochondrial dysfunction and ROS accumulation [41–44]. Collectively, these results suggest that *Eif2s2* deletion decreases the translation of mitochondrial fission proteins and then triggers ROS accumulation and apoptosis. Furthermore, the increased DNA damage and apoptosis, as well as the downregulated levels of RAD51 (essential for homologous recombination repair) were also observed in the *Eif2s2* deletion oocytes. Previous research has shown that excessive ROS can induce DNA damage in retinal precursor cell line R28 [45], GC-1 [46]and breast cancer cells [47]. DNA damage is typically repaired by homologous recombination and non-homologous end joining [48, 49]. If DNA damage is not repaired promptly, cells induce CHK2-p53-BAX-related pathways leading to G2/M phase arrest and cell apoptosis [50–52]. Therefore, these findings suggest that the mitochondrial dysfunction and ROS accumulation induced by *Eif2s2* deletion activate DNA damage, ultimately leading to oocyte apoptosis. In addition, the other subunits of the TC were also investigated. The deletion of *Eif2s1* and *Eif2s2* in oocytes showed similar phenotypes, and we primarily focus on the underlying mechanism of eIF2β in the present study. Future research should investigate whether eIF2α shares a similar mechanism with eIF2β in follicle development. These findings are consistent with our recent study reports that the deletion of *Eif2s2* in premeiotic germ cells led to oocyte arrest at the early diplotene stage and primordial follicle formation failure [30], and *Eif5* depletion in oocytes causes apoptosis through ROS accumulation and DNA damage [31].

Our research indicates that the secondary follicles and antral follicles were observed in GcKO mice and ZcKO mice, respectively, suggesting that the follicles with *Eif2s1* and *Eif2s2* deletion still develop into advanced stages. The possible reason is that the residual eIF2α and eIF2β (as shown in immunofluorescence staining and proteomic analysis data) maintain follicle development in *Eif2s1* and *Eif2s2* cKO mice. In addition, the decreased ubiquitination levels are also beneficial for slowing down the degradation of eIF2α and eIF2β, as well as other proteins, to maintain oocyte growth and development in *Eif2s1* and *Eif2s2* cKO mice. On the other hand, the moderate activation of ISR also helps maintain oocyte growth and follicle development in *Eif2s1* and *Eif2s2* cKO mice, as reported in HEK293T cells [53] and human fibroblasts [54]. It was worth noting that the primordial follicles with eIF2α and eIF2β expression were also reduced simultaneously with growing follicle decrease in *Eif2s1*- and *Eif2s2*-ZcKO mice ovaries, which is similar to previous findings in *Mfn1*- and *Mfn2*-ZcKO mice [55, 56]. This indicates that the existence of growing follicles contributes positively to the survival of primordial follicles. AMH is generated by the granulosa cells of growing follicles and can inhibit the transition of primary follicles from primordial follicles to alleviate chemotherapy drug-induced POI in mice [40, 57, 58]. Our results showed that the apoptosis of growing follicles resulted in the loss of primordial follicles and AMH treatment ameliorated primordial follicle loss in *Eif2s2*-ZcKO mice, suggesting that granulosa cell-produced AMH is also involved in maintaining the survival of primordial follicles. Therefore, follicle development is a continuous and uninterrupted biological process throughout the female reproductive lifespan [59]. Interruption of the development of growing follicles will reduce AMH production and accelerate the depletion of the ovarian follicular reserve [60–62]. However, the protective effect of AMH on the primordial follicles of *Eif2s2*-ZcKO mice was confirmed by the short-term culture. This protective effect needs further validation through the intraperitoneal injection of *Eif2s2*-ZcKO mice.

The depletion of translation initiation factor *Eif4e1b* in primary oocytes causes impairment of oocyte developmental competence [26]. The depletion of the translation regulatory factor *Zar1/2* in germ cells leads to defects in oocyte maturation [27]. In the present study, depletion of *Eif2s1* and *Eif2s2* in primary oocytes resulted in arrest at the earlier stage of growing follicles. The possible reason is that the depletion of *Eif2s1* and *Eif2s2* downregulates the global protein translation, while the depletion of *Zar1/2* and *Eif4e1b* impairs the selective mRNA translation in oocytes [27, 63]. These results suggest that deficiencies in different translation regulators lead to various reproductive phenotypes.

Recent research in clinical settings has found that individuals with Turner syndrome exhibit a deficiency in a cluster of oogonia characterized by high *EIF2S2* levels and germ cell apoptosis at 12–13 weeks following conception, subsequently developing POI after birth [64]. The findings from the present study and clinical reports suggest that the deficiency in the eIF2 subunits may result in POI. Oxidative stress induces the ISR, which is beneficial for restoring cellular homeostasis [65]. However, the excessive activation of the ISR can cause cell apoptosis [66]. Thus, drugs that prevent ISR overreaction are used to treat various diseases such as cardiovascular disease, cancer, and neurodegenerative diseases [66, 67]. A recent study has shown that the *EIF2B5* mutation causes human granulosa cells apoptosis by oxidative stress, and treatment with N-Acetylcysteine (an antioxidant) could alleviate granulosa cell apoptosis [68]. In *Eif2s1* and *Eif2s2* knockout mice, oxidative stress-induced ISR is the main cause of follicle development defects. Therefore, we propose that antioxidant treatment and ISR suppression could be potential therapeutic strategies for patients with eIF2 subunit mutants.

In conclusion, our study demonstrates that the deletion of *Eif2s1* and *Eif2s2* leads to the arrest of early growing follicle development and oocyte apoptosis. The mechanism involves the downregulated translation of mitochondrial fission regulatory factors and DNA damage repair proteins, as well as the upregulated levels of ROS and DNA damage. These findings provide valuable insights into the pathogenesis, genetic diagnosis, and potential therapeutic targets of POI.

## Supplementary information

Supplementary Figures and Tables

Original data

## Data Availability

Raw proteomic data have been deposited in the ProteomeXchange under accession number PXD054478. RNA-seq data have been deposited in the NCBI Sequence Read Archive (SRA) database under accession number PRJNA1142762. All data supporting the findings of this study are available within the article and/or the supplementary information. Additional data related to this paper may be requested from the authors.
