# Supplementary material for: Oocyte-specific knockout of eIF2 subunits causes apoptosis of mouse oocytes within the early growing follicles via mitochondrial dysfunctions and DNA damage
Source: Cell Death Dis. 2026 Feb 2;17(1):196. doi: 10.1038/s41419-026-08449-y (PMC12877101; doi:10.1038/s41419-026-08449-y)
Supplement: Supplementary file 1 — Supplementary Figures and Tables [file 41419_2026_8449_MOESM1_ESM.docx]

**Supplementary figures and tables**

**
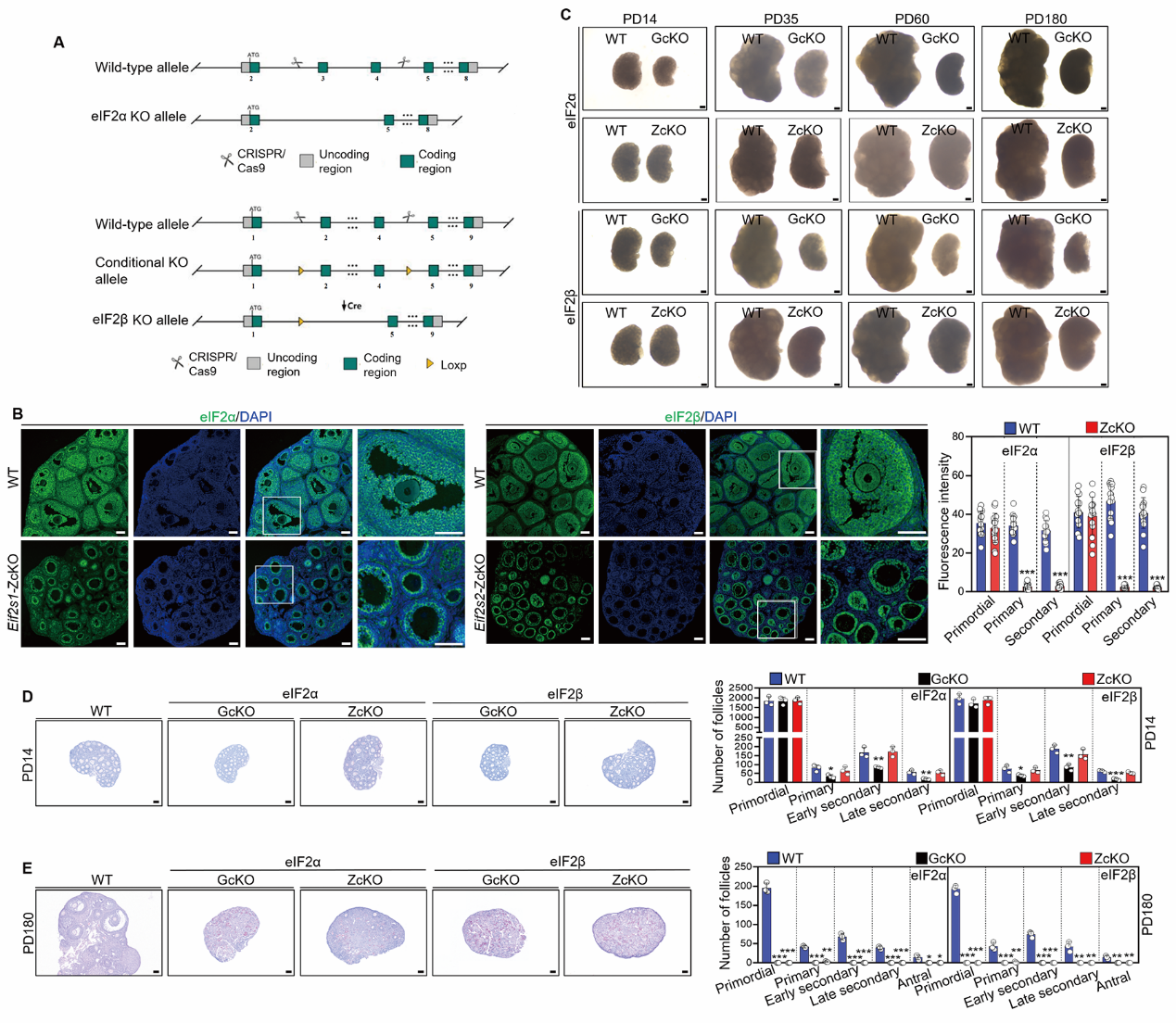
**

**Supplementary Fig. 1 *Eif2s1 and 2* deletion in oocytes impairs follicle development. A** Schematic representation of *Eif2s1 and 2* deletion by *Gdf9*-Cre and *Zp3*-Cre-mediated recombinase in oocytes. **B** Immunofluorescence staining of eIF2α and eIF2β in WT, *Eif2s1*- and *Eif2s2*-ZcKO ovaries at PD21. Quantiﬁcation of eIF2α and eIF2β fluorescence intensity in the oocytes within different follicles, n = 15 oocytes from 3 females. **C** Representative images of ovaries isolated from WT, GcKO and ZcKO female mice at the indicated age. **D, E** PAS staining showing ovarian histology of WT, GcKO and ZcKO mice at the indicated ages. Quantiﬁcation of the number of follicles at different developmental stages, n = 3 females for each genotype. In each experiment, n ≥ 3 biological replicates. Bars indicate the mean ± SD. A two-sided Student’s t-test was used to determine *P* values. (**P* < 0.05, ***P* < 0.01 and ****P* < 0.001). Scale bar: 100 μm.

**
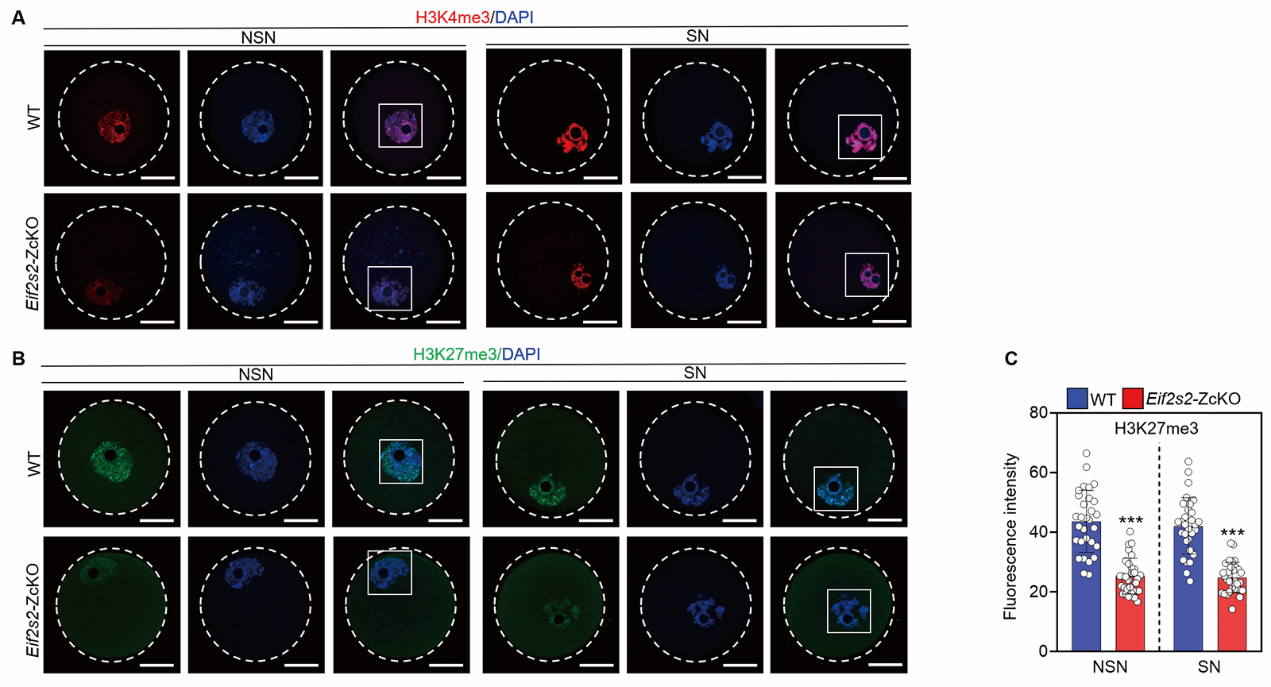
**

**Supplementary Fig. 2 *Eif2s2* deletion in oocytes impairs histone methylation in oocytes.** **A, B** Immunofluorescence staining of H3K4me3 and H3K27me3 in WT and ZckO oocytes. **C** Quantiﬁcation of H3K27me3 fluorescence intensity (n = 30 oocytes). In each experiment, n ≥ 3 biological replicates. Bars indicate the mean ± SD. A two-sided Student’s t-test was used to determine *P* values. (****P* < 0.001). Scale bars: 25 μm.

**
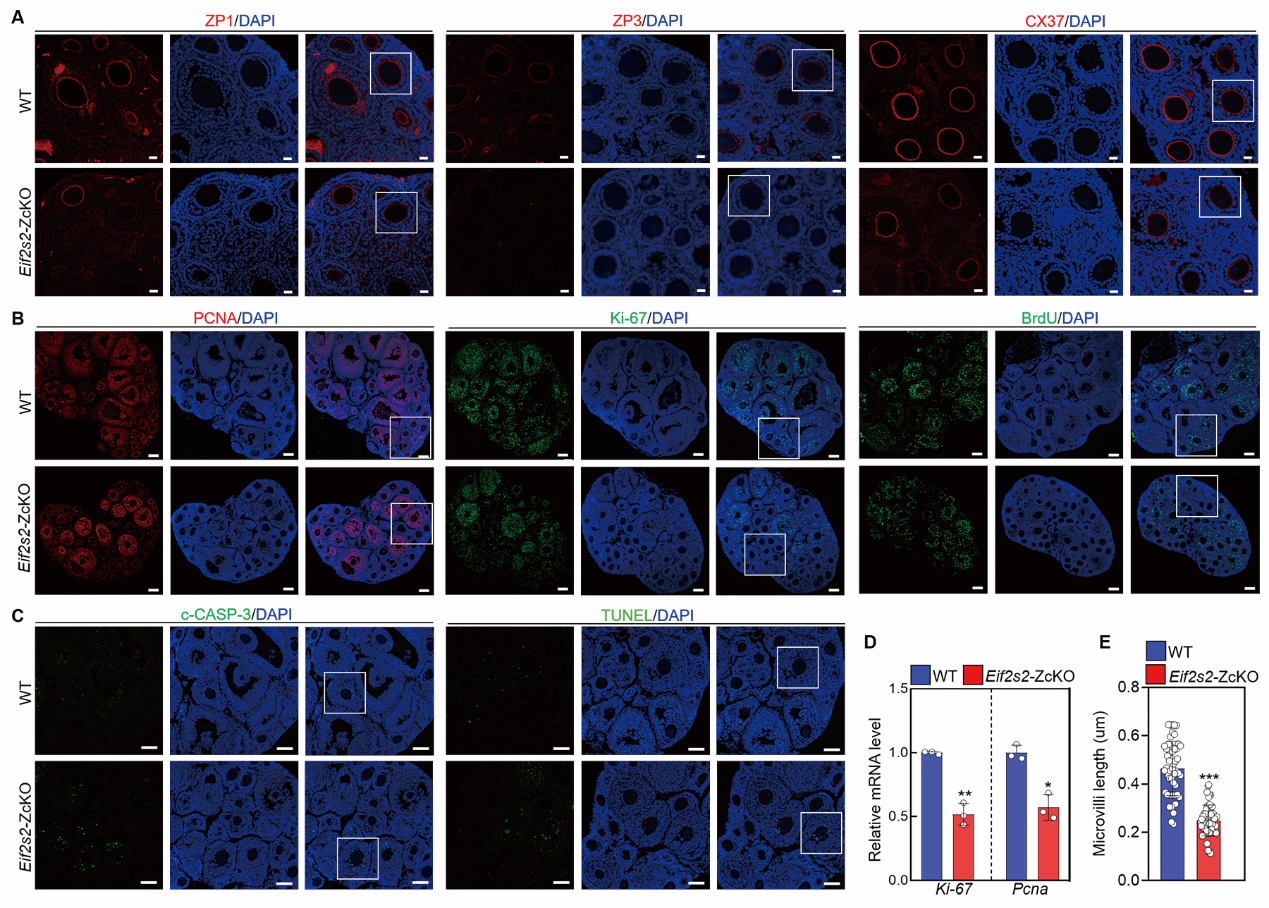
**

**Supplementary Fig. 3 *Eif2s2* deletion in oocytes impairs the bidirectional communication between the oocyte and granulosa cells. A** Immunofluorescence staining of CX37, ZP1 and ZP3 in WT and ZcKO ovaries at PD21. **B** Immunofluorescence staining of Ki-67, PCNA and BrdU in WT and ZcKO ovaries at PD21. **C** Immunofluorescence staining of TUNEL and c-CASP-3 (cleaved Caspase-3) in WT and ZcKO ovaries at PD21. **D** qPCR assay showing the relative expression levels of *Ki-67* and *Pcna* mRNA in WT and ZcKO granulosa cells. **E** Quantiﬁcation of the length of microvilli in WT and *Eif2s2*-ZcKO oocytes. Four oocytes (at least 50 microvillis) were used in each group. In each experiment, n ≥ 3 biological replicates. Bars indicate the mean ± SD. A two-sided Student’s t-test was used to determine *P* values. (**P* < 0.05, ***P* < 0.01 and ****P* < 0.001). Scale bars: 25 μm (A) and 100 μm (B, C).

**
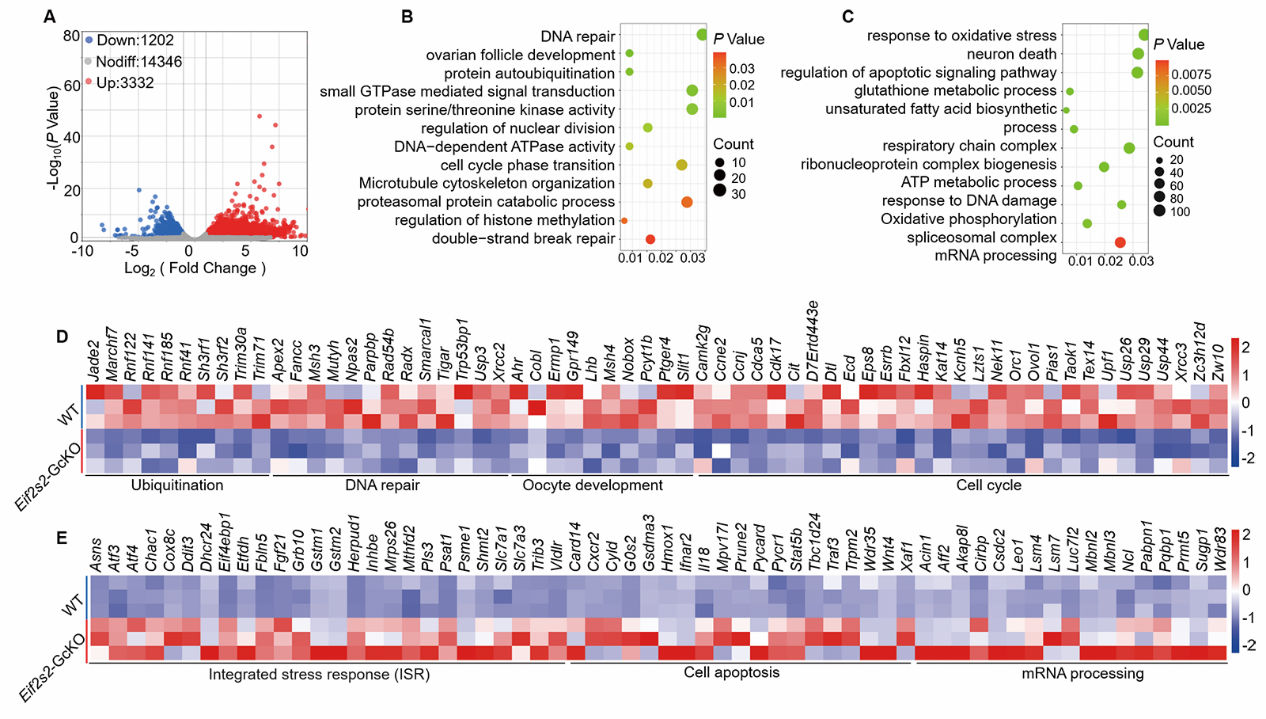
**

**Supplementary Fig. 4 *Eif2s2* deletion in oocytes impairs the integrity of the transcriptome.** **A** Volcano plot illustrating the differentially expressed transcripts in WT and ZcKO oocytes. **B, C** Bubble chart illustrating the enriched GO terms associated with the significantly downregulated and upregulated transcripts in GcKO oocytes. **D, E** Heatmaps illustrating downregulation and upregulation between WT and GcKO oocytes in the expression of a group of transcripts involved in various processes. In each experiment, n ≥ 3 biological replicates.

**
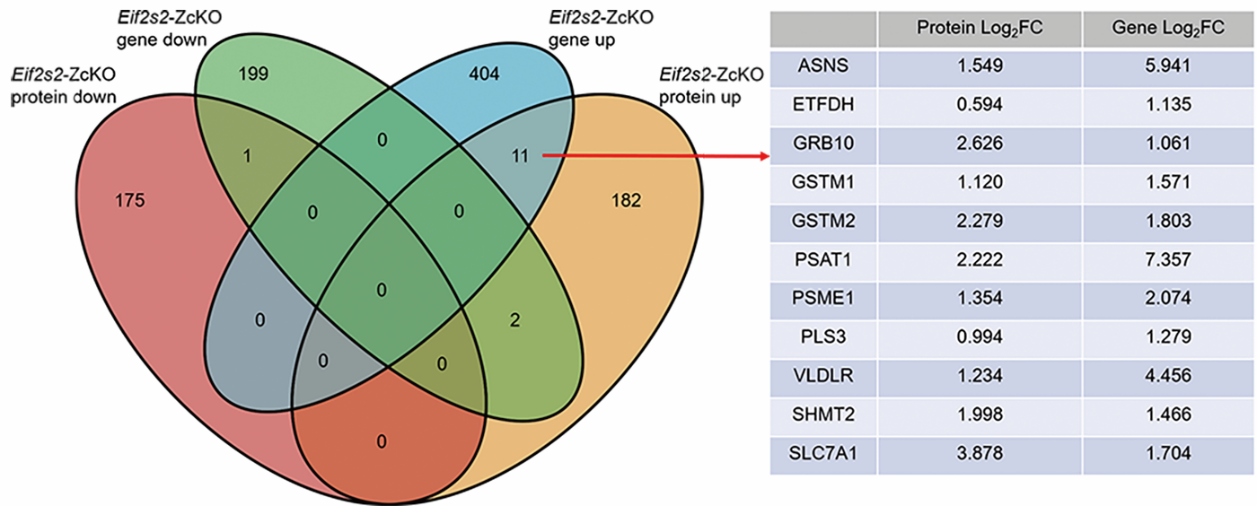
**

**Supplementary Fig. 5 The integration analysis between transcriptomic and proteomic in ZcKO oocytes.** Venn diagram illustrating the relationship of up-and down-regulated protein and gene identified in *Eif2s2*-ZcKO oocytes.

**
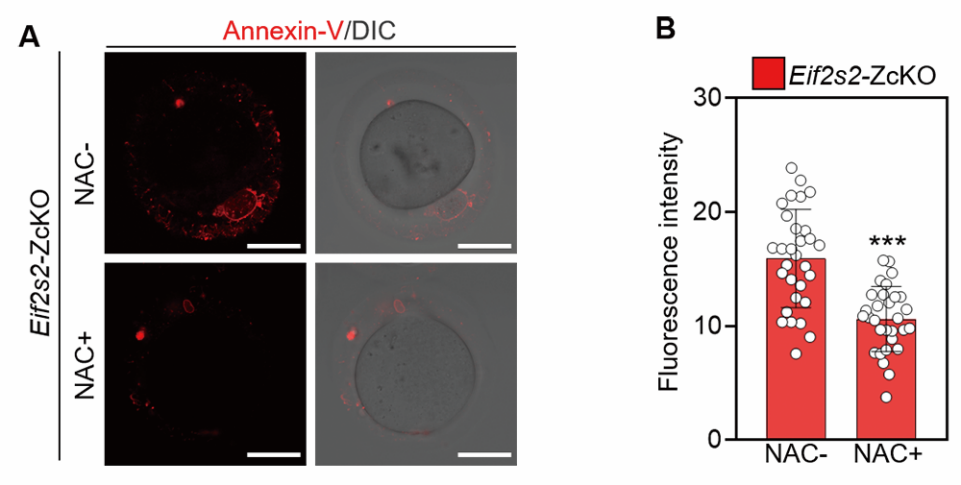
**

**Supplementary Fig. 6 Annexin V staining. A** Representative images of Annexin V staining in *Eif2s2*-ZckO oocytes with (NAC+) or without NAC (NAC-) treatment. **B** Quantiﬁcation of Annexin V fluorescence intensity of in *Eif2s2*-ZckO oocytes (n = 30). Bars indicate the mean ± SD. A two-sided Student’s t-test was used to determine *P* values. (****P* < 0.001). Scale bars: 25 μm.

**
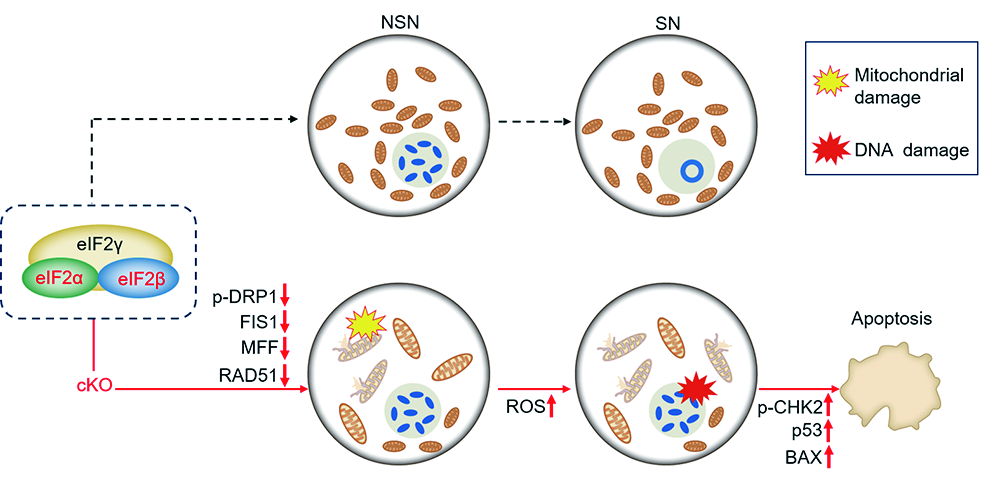
**

**Supplementary Fig. 7 Schematic diagram of eIF2 subunits involved in translation initiation and oocyte growth.** Oocyte growth requires massive protein synthesis. eIF2 submits contribute to oocyte growth by promoting protein translation. Deletion of *Eif2s1* and *2* in mouse oocytes impairs the translation of mitochondrial fission-related proteins (p-DRP1, FIS1 and MFF) and DNA damage repair protein (RAD51), followed by mitochondrial damage. Subsequently, excessive ROS-induced DNA damage triggers the CHK2-p53-BAX pathway, resulting in the apoptosis of oocytes.

**Table S1. Primers for PCR**

| Primer name | Genes targeted | Application | Sequences (5’-3’) |
| --- | --- | --- | --- |
| FRT-F | *Eif2s1* | Identification of *Eif2s1* flox | GGACTTCGTTGTTAAAGGCTTAGG |
| FRT-R |  |  | CCAGGCTCATTCAAACTTATCACCGC |
| FRT-F | *Eif2s2* | Identification of *Eif2s2* flox | TGCTCCAATGTGCTTTCGATCA |
| FRT-R |  |  | GGAACCCTATCAGCAACCCTTAC |
| Cre-R1 | *Gdf9*-Cre | Identification of *Gdf9*-Cre | AAGAACCTGATGGACATGTTCAG |
| Cre-R2 |  |  | CTGATTCTGGCAATTTCGG |
| Cre-R1 | *Zp3*-Cre | Identification of *Zp3*-Cre | CAGATGAGGTTTGAGGCCACAG |
| Cre-R2 |  |  | TTCTTGCGAACCTCATCACTC |

**Table S2. Primers for qRT-PCR**

| Genes | Forward primers (5’-3’) | Reverse primers (5’-3’) | |
| --- | --- | --- | --- |
| *Eif2s1* | ATGCCGGGGCTAAGTTGTAGA | AACGGATACGTCGTCTGGATA | |
| *Eif2s2* | TCCACAGGTCGTCCGAGTAG | ACCACTTGTACCCAATTCTGC | |
| *Ki-67* | CATTGACCGCTCCTTTAGGT | TCGCCTTGATGGTTCCT | |
| *Pcna* | TTTGAGGCACGCCTGATCC | GGAGACGTGAGACGAGTCCAT | |
| *Asns* | GCAGTGTCTGAGTGCGATGAA | TCTTATCGGCTGCATTCCAAAC | |
| *Atf3* | CCCCTGGAGATGTCAGTCAC | CGGTGCAGGTTGAGCATGT | |
| *Atf4* | CCTATAAAGGCTTGCGGCCA | CACTGCTGCTGGATTTCGTG | |
| *Chac1* | CTGTGGATTTTCGGGTACGG | CCCCTATGGAAGGTGTCTCC | |
| *Ddit3* | CTGGAAGCCTGGTATGAGGAT | CAGGGTCAAGAGTAGTGAAGGT | |
| *Eif4e1b* | GGGGACTACAGCACCACTC | CTCATCGCTGGTAGGGCTA | |
| *Fgf21* | CTGCTGGGGGTCTACCAAG | CTGCGCCTACCACTGTTCC |  |
| *Herpud1* | GCAGTTGGAGTGTGAGTCG | TCTGTGGATTCAGCACCCTTT |  |
| *Hmox1* | AGGTACACATCCAAGCCGAGA | CATCACAGCTTAAAGCCTTCT |  |
| *Inhbe* | CTAACCAGCCGTCCCAGAATA | GTGCCCGGAAAAGAGGGAG |  |
| *Mrps26* | CATGACCCGCCTGCCAAAT | TCTCCCGGTACTGTCGGTAG |  |
| *Mthfd2* | AGTGCGAAATGAAGCCGTTG | GACTGGCGGGATTGTCACC |  |
| *Slc7a3* | TGAGCACCCTCGACTTAGTG | CACAATGGATGGTCCTGCTTTA |  |
| *Trib3* | ACCTTCAGAGCGACTTGTGGG | GCTTGGCCCAAAAAGTCAGG |  |
| *Rpl19* | CTGAAGGTCAAAGGGAATGTGTTC | TGGTCAGCCAGGAGCTTCTTG |  |

**Table S3. List of primary antibodies used in immune detection**

| Antibody | Catalog Code | | Source | | Host | | | Dilution | |
| --- | --- | --- | --- | --- | --- | --- | --- | --- | --- |
|  |  |  |  |  |  |  |  | IF | WB |
| AURKA | | A2121 | | Abclonal | | Rabbit | — | | 1:1000 |
| BMP15 | | 18982-1-AP | | Proteintech | | Rabbit | 1:100 | | 1:1000 |
| BAX | | 50599-2-Ig | | Proteintech | | Rabbit | 1:200 | | 1:1000 |
| BCL-xL | | 2764 | | CST | | Rabbit | 1:200 | | 1:1500 |
| BrdU | | ab1893 | | Abcam | | Sheep | 1:200 | | — |
| CDC25B | | sc-56266 | | Santa Cruz | | Mouse | — | | 1:500 |
| CX37 | | CX37A11-A | | Alpha Diagnostic International | | Rabbit | 1:100 | | 1:1000 |
| Cleaved Caspase-3 | | 9664 | | CST | | Rabbit | 1:50 | | — |
| DDX4 | | ab27591 | | Abcam | | Mouse | 1:200 | | 1:1000 |
| DRP1 | | A2586 | | Abclonal | | Rabbit | — | | 1:1000 |
| Phospho-DRP1-S616 | | AP1353 | | Abclonal | | Rabbit | 1:200 | | 1:1000 |
| eIF2B1 | | A7892 | | Abclonal | | Rabbit | — | | 1:1000 |
| eIF2α | | 82936-1-RR | | Proteintech | | Rabbit | 1:200 | | 1:1000 |
| eIF2β | | 68463-1-Ig | | Proteintech | | Rabbit | 1:200 | | 1:1000 |
| FIS1 | | sc-376447 | | Santa Cruz | | Mouse | 1:100 | | 1:1000 |
| GDF9 | | ab254323 | | Abcam | | Rabbit | 1:200 | | 1:1000 |
| H3K4me3 | | ab8580 | | Abcam | | Rabbit | 1:200 | | — |
| H3K27me3 | | P37961-3F | | Abmart | | Rabbit | 1:200 | | — |
| Ki-67 | | 9129s | | CST | | Rabbit | 1:200 | | — |
| MFF | | sc-398617 | | Santa Cruz | | Mouse | 1:100 | | 1:500 |
| p53 | | sc-126 | | Santa Cruz | | Rabbit | 1:100 | | — |
| PCNA | | 2586 | | CST | | Mouse | 1:200 | | 1:1000 |
| Puromycin | | A23031 | | Abclonal | | Rabbit | — | | 1:1000 |
| Phospho-CHK2-T68 | | HY-80799 | | MCE | | Rabbit | 1:200 | | — |
| RAD51 | | ab133534 | | Abcam | | Rabbit | — | | 1:1000 |
| TOMM20 | | A19403 | | Abclonal | | Rabbit | 1:200 | | — |
| ZP1 | sc-32751 | | | Santa Cruz | | Mouse | 1:100 | | — |
| ZP3 | sc-398359 | | | Santa Cruz | | Mouse | 1:100 | | — |
| α-tubulin | ab195887 | | | Abcam | | — | 1:400 | | — |
| γH2AX | ab206900 | | | Abcam | | Mouse | — | | 1:500 |
| γH2AX | ab22551 | | | Abcam | | — | 1:300 | | — |
| β-actin | 4967 | | | CST | | Rabbit | — | | 1:1000 |

IF: Immunofluorescence; WB: Western blotting

**Table S4. Primers for quantification of mtDNA copy number**

| Genes | Forward primers (5’-3’) | Reverse primers (5’-3’) |
| --- | --- | --- |
| *ND1* | CTAGCAGAAACAAACCGGGC | CCGGCTGCGTATTCTACGTT |
| *HK2* | GCCAGCCTCTCCTGATTTTAGTGT | GGGAACACAAAAGACCTCTTCTGG |
