# Supplementary figures and images for: Oocyte-specific knockout of eIF2 subunits causes apoptosis of mouse oocytes within the early growing follicles via mitochondrial dysfunctions and DNA damage
Source: Cell Death Dis. 2026 Feb 2;17(1):196. doi: 10.1038/s41419-026-08449-y (PMC12877101; doi:10.1038/s41419-026-08449-y)

**Fig 1E.**

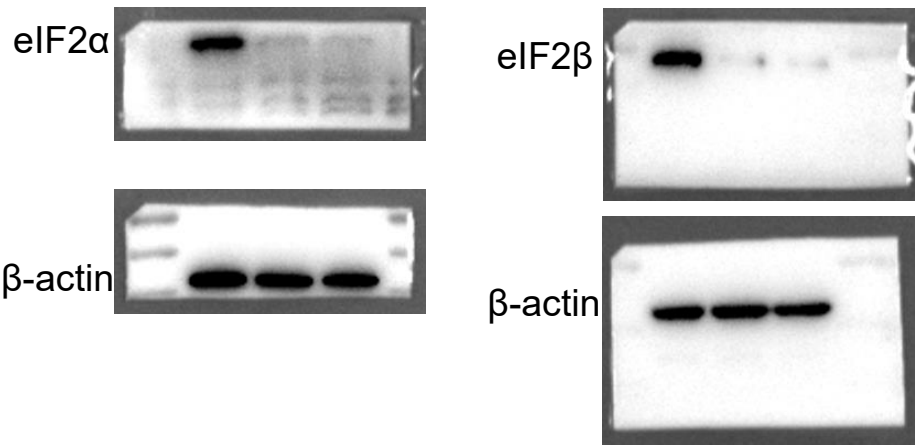

**Fig 3C.**

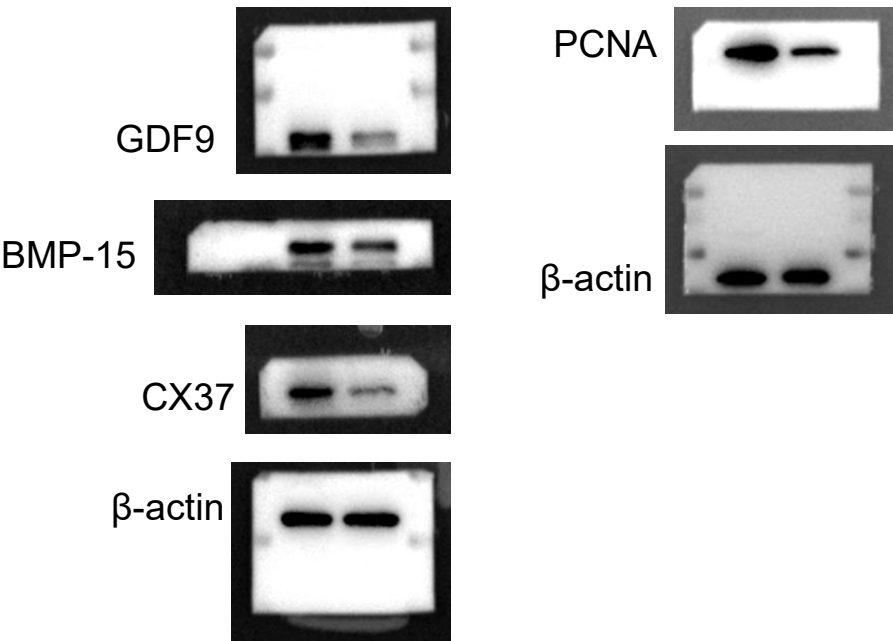

**Fig 4A.**

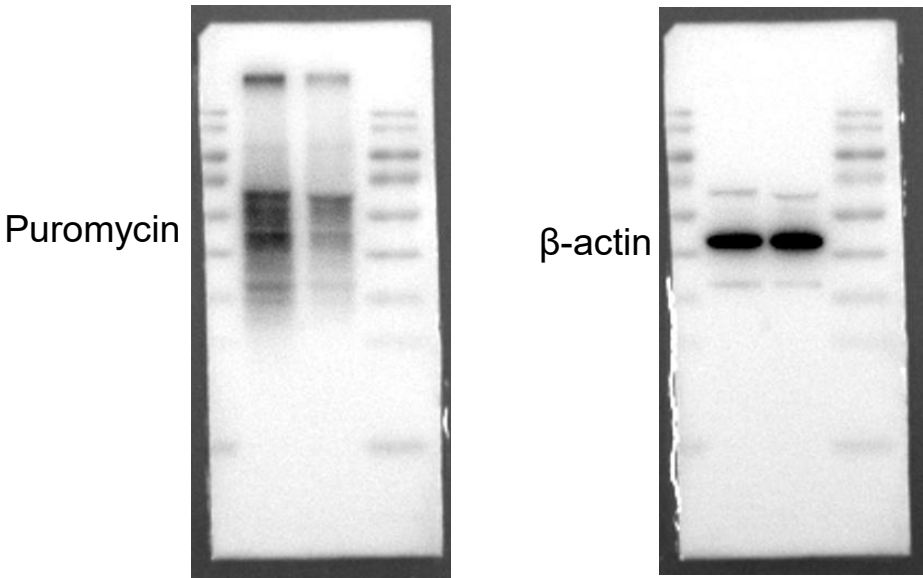

**Fig 4D.**

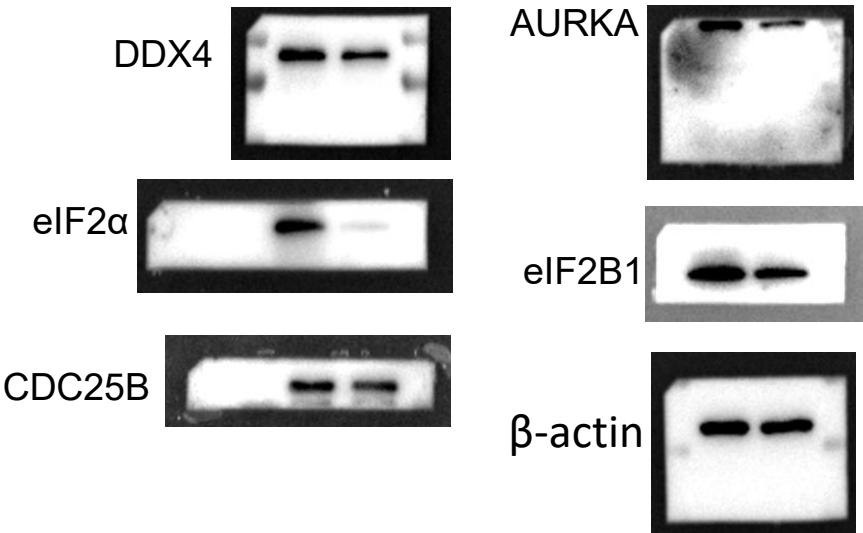

**Fig 5F.**

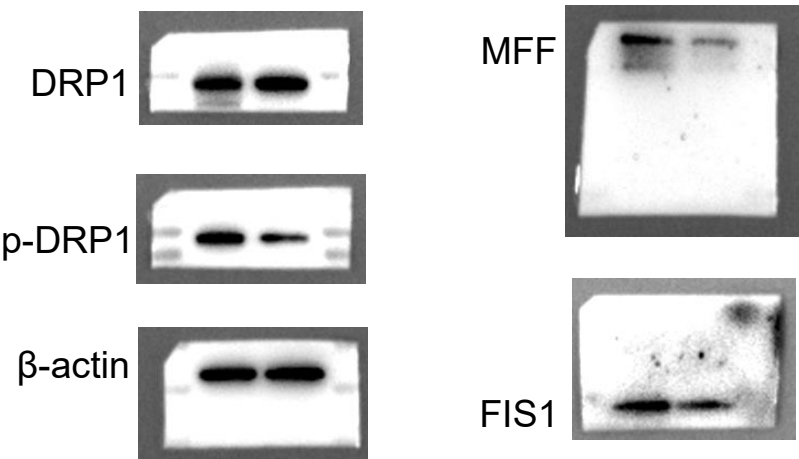

**Fig 8D.**

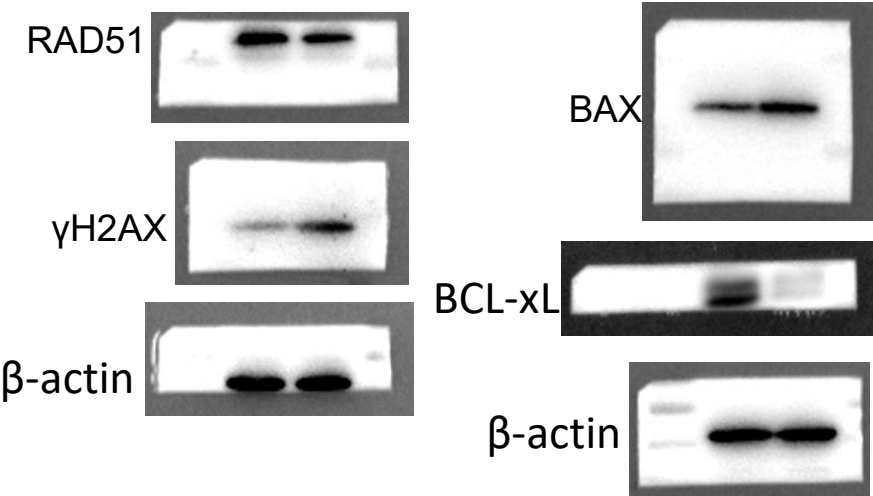

Supplement: Supplementary file 2 — Original data [file 41419_2026_8449_MOESM2_ESM.pdf]
